# Supplementary figures and images for: Pharmacokinetic profiles of Moutan Cortex after single and repeated administration in a dinitrobenzene sulfonic acid-induced colitis model
Source: PLoS One. 2025 Dec 2;20(12):e0337713. doi: 10.1371/journal.pone.0337713 (PMC12671744; doi:10.1371/journal.pone.0337713)

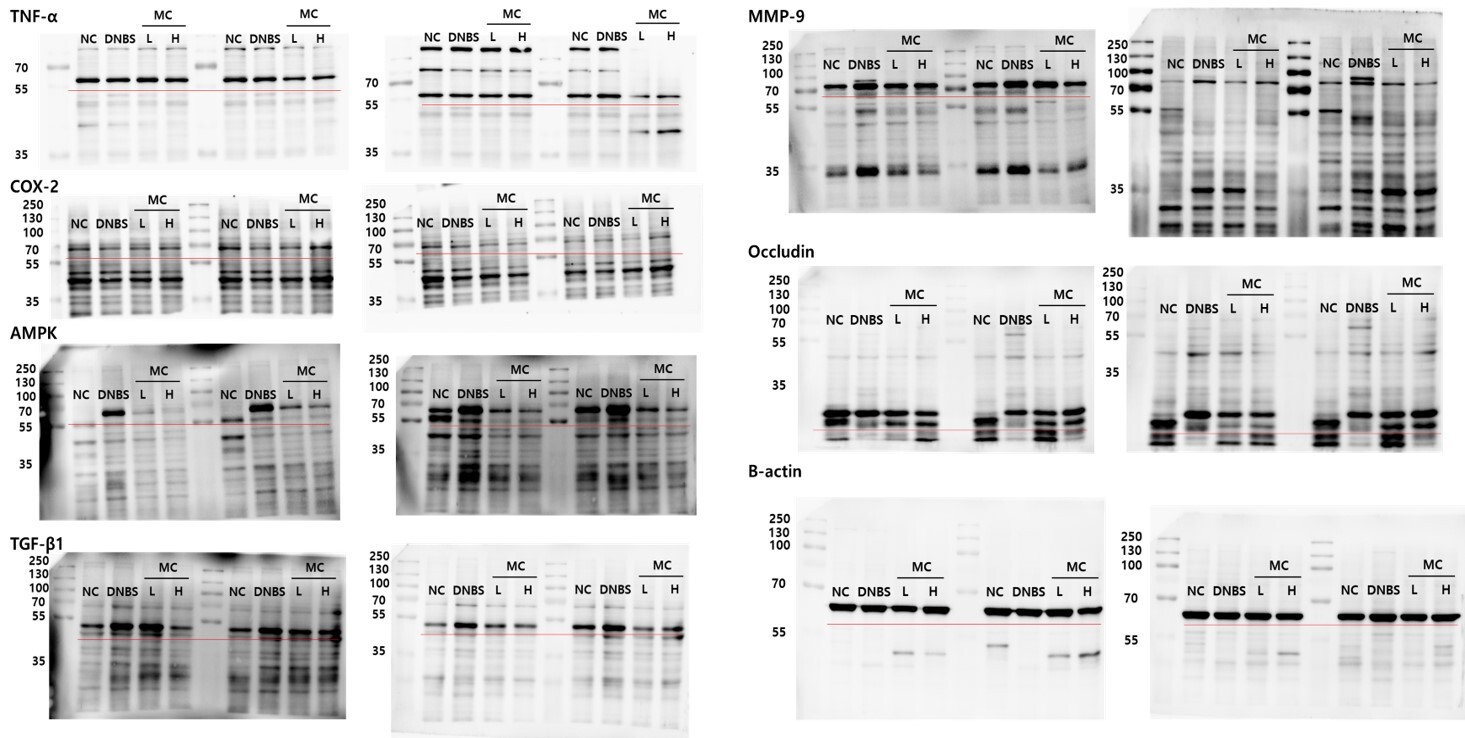

Supplement: S1 Fig — AMPK, AMP-activated protein kinase; COX-2, cyclooxygenase-2; MMP-9, matrix metallopeptidase-9; TGF-β1, transforming growth factor-beta 1; TNF-α, tumor necrosis factor-alpha. (JPG) [file pone.0337713.s001.jpg]

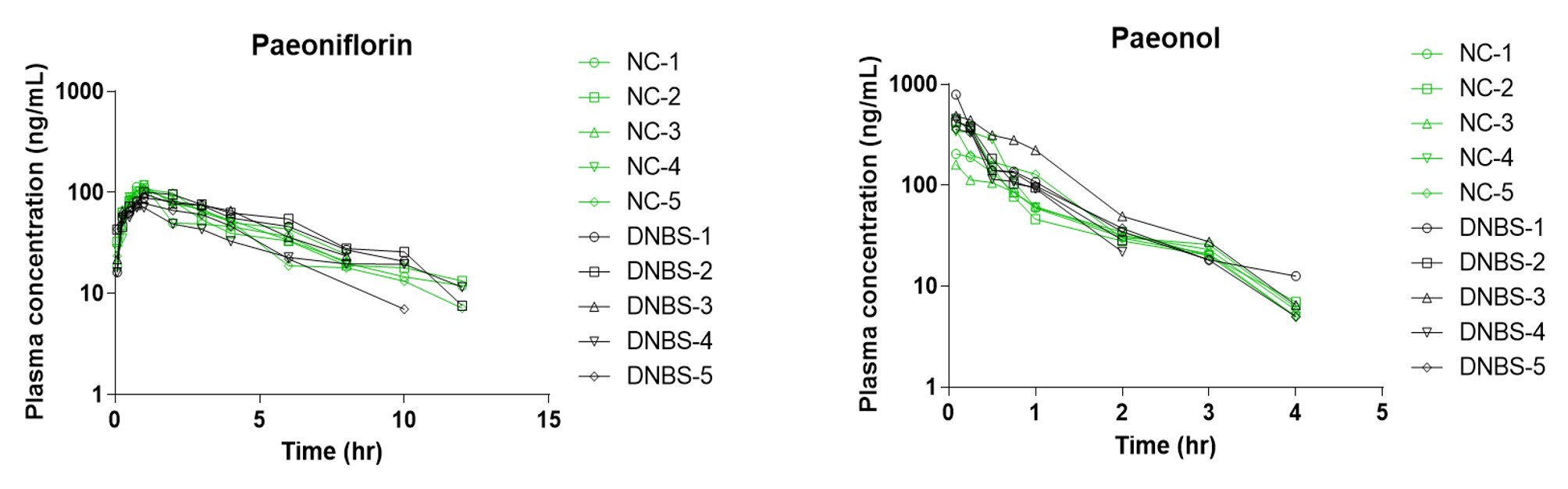

Supplement: S2 Fig — Individual plasma concentration-time curves for paeoniflorin and paeonol following a single oral administration of MC (2.5 g/kg) in NC and DNBS-induced colitis rats (n = 5). DNBS, dinitrobenzene sulfonic acid; MC, Moutan Cortex; NC, normal control. (JPG) [file pone.0337713.s002.jpg]

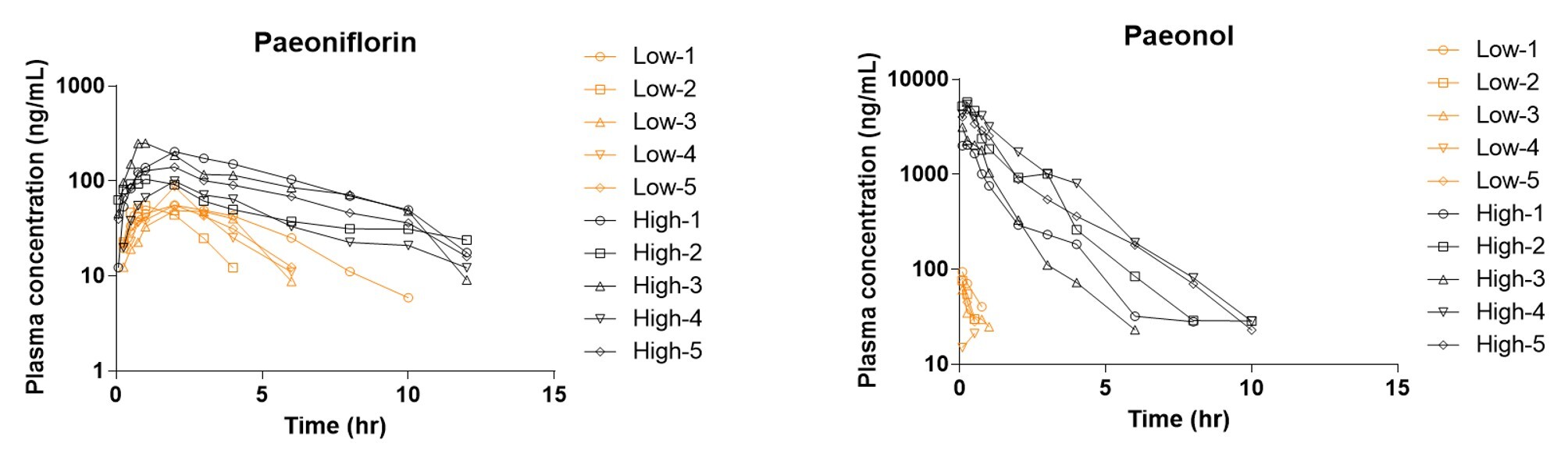

Supplement: S3 Fig — Individual plasma concentration-time profiles for paeoniflorin and paeonol after repeated oral dosing for six consecutive days with low (0.5 g/kg) or high (2.5 g/kg) MC in DNBS-treated rats (n = 5). DNBS, dinitrobenzene sulfonic acid; MC, Moutan Cortex. (JPG) [file pone.0337713.s003.jpg]
