# Supplementary material for: Pharmacokinetic profiles of Moutan Cortex after single and repeated administration in a dinitrobenzene sulfonic acid-induced colitis model
Source: PLoS One. 2025 Dec 2;20(12):e0337713. doi: 10.1371/journal.pone.0337713 (PMC12671744; doi:10.1371/journal.pone.0337713)
Supplement: S1 Table — CV, coefficient of variation; LOD, limit of detection; LOQ, limit of quantification; QC, quality control. (DOCX) [file pone.0337713.s004.docx]

| **Analyte** | **QC (ng/mL)** | **Day** | **Recovery** | | | **Inter-day** | | **Intra-day** | |
| --- | --- | --- | --- | --- | --- | --- | --- | --- | --- |
|  |  |  | **1** | **2** | **3** | **Mean recovery (%)** | **CV (%)** | **Mean recovery (%)** | **CV (%)** |
| **Paeonol** | 100 | 1 | 97.6 | 97.1 | 97.4 | 97.4 | 0.3 | 99.8 | 3.4 |
|  |  | 2 | 95.3 | 105.8 | 93.7 | 98.3 | 6.6 |  |  |
|  |  | 3 | 103.5 | 97.8 | 109.6 | 103.6 | 5.9 |  |  |
|  | 500 | 1 | 122.7 | 125.8 | 123.5 | 124.0 | 1.6 | 124.5 | 0.8 |
|  |  | 2 | 124.2 | 123.3 | 124.6 | 124.0 | 0.7 |  |  |
|  |  | 3 | 124.2 | 127.5 | 124.3 | 125.3 | 1.9 |  |  |
|  | 1000 | 1 | 94.4 | 93.7 | 94.2 | 94.1 | 0.4 | 94.8 | 1.4 |
| **LOD (ng/mL): 1** |  | 2 | 94.1 | 101.1 | 94.0 | 96.4 | 4.1 |  |  |
| **LOQ (ng/mL): 3** |  | 3 | 94.0 | 93.3 | 94.0 | 93.8 | 0.4 |  |  |
| **Paeoniflorin** | 100 | 1 | 94.3 | 98.9 | 96.2 | 96.5 | 2.3 | 95.9 | 1.0 |
|  |  | 2 | 94.7 | 93.4 | 95.9 | 94.7 | 1.3 |  |  |
|  |  | 3 | 92.6 | 98.2 | 98.5 | 96.4 | 3.3 |  |  |
|  | 500 | 1 | 119.5 | 123.7 | 122.6 | 121.9 | 2.2 | 122.1 | 1.3 |
|  |  | 2 | 121.2 | 121.3 | 120.3 | 120.9 | 0.6 |  |  |
|  |  | 3 | 124.1 | 122.8 | 123.4 | 123.4 | 0.6 |  |  |
|  | 1000 | 1 | 95.3 | 94.2 | 94.5 | 94.6 | 0.6 | 94.6 | 0.3 |
| **LOD (ng/mL): 1** |  | 2 | 94.8 | 94.8 | 95.1 | 94.9 | 0.1 |  |  |
| **LOQ (ng/mL): 3** |  | 3 | 94.2 | 94.4 | 94.3 | 94.3 | 0.1 |  |  |

**Supplementary Table 1. Validation results for the analysis method of paeonol and paeoniflorin.**

CV, coefficient of variation; LOD, limit of detection; LOQ, limit of quantification; QC, quality control;
