# Supplementary material for: Pharmacokinetic profiles of Moutan Cortex after single and repeated administration in a dinitrobenzene sulfonic acid-induced colitis model
Source: PLoS One. 2025 Dec 2;20(12):e0337713. doi: 10.1371/journal.pone.0337713 (PMC12671744; doi:10.1371/journal.pone.0337713)
Supplement: Attachment — Submitted filename: Response to Reviewers.docx [file pone.0337713.s006.docx]

**Reviewer 1.**

1) In the Abstract section, the authors provided some results with fold. If the authors can provide number comparisons such as “number vs. number”, will help readers understand the work of the manuscript more directedly.

We agree with this comment. To help readers understand the differences more intuitively, we revised the text to include the exact numerical values instead of fold changes (Line 34–35 and 37–39).

2) In the Keywords section, the last keyword should be deleted. And the word “pharmacokinetic” should be moved as the last keywords.

We appreciate the thoughtful suggestion. We have removed the last key word and repositioned “pharmacokinetic” as the final keyword.

3) In line 67 and ling 68, the abbreviation ADME should be added.

Thank you for the comment. We have added the abbreviation “ADME” to the text (Line 68-69).

4) As the authors used animal experiments, ethical issues should be provided or exhibited.

Thank you for the valuable comment. We have provided detailed information on ethical approval and considerations with the laboratory animal care guidelines (Line 101-105).

5) When introducing the western blot analysis procedure, the version of the ImageJ software should be provided.

Thank you for pointing that out. We have added the ImageJ software version used for densitometric analysis in the Methods part (Line 209).

**Reviewer 2.**

1) The manuscript inconsistently refers to “mice” in some sections (abstract/introduction) while the Methods clearly state that Sprague-Dawley rats were used. This is a fundamental error and must be corrected throughout the text for clarity and accuracy.

Thank you for pointing this out. We carefully reviewed the entire manuscript and corrected all to accurately refer to rats instead of mice (Line 31).

2) Each group contains only five animals, which is relatively small for pharmacokinetic and histological comparisons, especially given the large interindividual variability observed (e.g., paeonol Cmax at high dose). The authors should either increase the sample size, provide power calculations, or at minimum show individual concentration–time profiles to illustrate variability.

We appreciate your concern. We agree that using five animals per group may limited in several PK endpoints. We have now included the individual concentration-time profiles for paeonol and paeoniflorin in the Supplementary figure (Fig. S2 and S3) and referred them in the text (Line 242 and 273-274). Additionally, we inserted a new paragraph in the manuscript (Line 426-432) to describe this inclusion.

3) Please provide raw PK data (machine-readable) as mentioned. Reference them clearly in the main text.

We appreciate the reviewer’s comment. The raw plasma concentration-time data for all individual animals have been provided in a machine-readable format thought the Zenodo database (DOI: 10.5281/zenodo.17462573). This information has been clearly referenced in the main text (Line 163-164).

Minor
1) Given the dramatic increase in paeonol systemic exposure at the high dose, safety/toxicity monitoring is important. The manuscript currently states that no euthanasia endpoints were met, but it does not report biochemical markers (e.g., liver/kidney function) or histopathological observations beyond the colon. These should be included or at least discussed as a limitation.

We agree with your suggestion. We have expanded the discussion of limitations to acknowledge that safety biomarkers (ALT, AST, ALP,…) and multi-organ histopathology were not assessed in this study, which limits definitive conclusions on systemic toxicity (Line 433-436).
